# Systemic therapy for metastatic renal cell carcinoma in the first-line setting: a systematic review and network meta-analysis

**DOI:** 10.1007/s00262-020-02684-8

**Published:** 2020-08-05

**Authors:** Keiichiro Mori, Hadi Mostafaei, Noriyoshi Miura, Pierre I. Karakiewicz, Stefano Luzzago, Manuela Schmidinger, Andreas Bruchbacher, Benjamin Pradere, Shin Egawa, Shahrokh F. Shariat

**Affiliations:** 1 Department of Urology, Medical University of Vienna, Währinger Gürtel 18-20, 1090 Vienna, Austria; 2 Department of Urology, The Jikei University School of Medicine, Tokyo, Japan; 3 Research Center for Evidence Based Medicine, Tabriz University of Medical Sciences, Tabriz, Iran; 4 Department of Urology, Ehime University Graduate School of Medicine, Ehime, Japan; 5 Cancer Prognostics and Health Outcomes Unit, University of Montreal Health Centre, Montreal, Canada; 6 Department of Urology, European Institute of Oncology, IRCCS, Milan, Italy; 7 Clinical Division of Oncology, Department of Medicine I and Comprehensive Cancer Center, Medical University of Vienna, Vienna, Austria; 8 Department of Urology, University Hospital of Tours, Tours, France; 9 Division of Urology, Department of Special Surgery, The University of Jordan, Amman, Jordan; 10 Department of Urology, Weill Cornell Medical College, New York, NY USA; 11 Department of Urology, University of Texas Southwestern, Dallas, TX USA; 12 Karl Landsteiner Institute of Urology and Andrology, Vienna, Austria; 13 Department of Urology, Second Faculty of Medicine, Charles University, Prague, Czech Republic; 14 Institute for Urology and Reproductive Health, I.M. Sechenov First Moscow State Medical University, Moscow, Russia; 15 European Association of Urology Research Foundation, Arnhem, Netherlands; 16 Université François Rabelais de Tours, PRES Centre Val de Loire, Tours, France

**Keywords:** Renal cell carcinoma, Network meta-analysis, First-line, Immune-checkpoint inhibitors

## Abstract

**Purpose:**

Management of metastatic renal cell cancer (mRCC) has undergone a paradigm shift with immune-checkpoint inhibitors (ICI) in the first-line setting. However, direct comparative data are inadequate to inform treatment decisions. Therefore, we aimed to assess first-line therapy for mRCC and indirectly compare the efficacy and safety of currently available treatments.

**Materials and methods:**

Multiple databases were searched for articles published before June 2020. Studies that compared overall and/or progression-free survival (OS/PFS) and/or adverse events (AEs) in mRCC patients were considered eligible.

**Results:**

Six studies matched our eligibility criteria. For OS, pembrolizumab plus axitinib [hazard ratio (HR) 0.85, 95% credible interval (CrI) 0.73–0.98] and nivolumab plus ipilimumab (HR 0.86, 95% CrI 0.75–0.99) were significantly more effective than sunitinib, and pembrolizumab plus axitinib was probably the best option based on analysis of the treatment ranking. For PFS, pembrolizumab plus axitinib (HR 0.86, 95% CrI 0.76–0.97) and avelumab plus axitinib (HR 0.85, 95% CrI 0.74–0.98) were statistically superior to sunitinib, and avelumab plus axitinib was likely to be the preferred option based on analysis of the treatment ranking, closely followed by pembrolizumab plus axitinib. Nivolumab plus ipilimumab had significantly lower rates of serious AEs than sunitinib.

**Conclusion:**

Pembrolizumab plus axitinib seemed to be the most efficacious first-line agents, while nivolumab plus ipilimumab had the most favorable efficacy–tolerability equilibrium. These findings may facilitate individualized treatment strategies and inform future direct comparative trials in an expanding treatment options without direct comparison between approved drugs.

**Electronic supplementary material:**

The online version of this article (10.1007/s00262-020-02684-8) contains supplementary material, which is available to authorized users.

## Introduction

Renal cell carcinoma (RCC) is among the top 10 most frequently diagnosed cancers worldwide [1]. Approximately 25% of patients with RCC who present with metastatic tumors at the time of initial diagnosis typically require systemic treatment. Moreover, another 20–50% of RCC patients with localized disease eventually develop metastatic RCC (mRCC) [2–4]. Targeted therapies with lesser toxicity and higher survival benefits have become the mainstay of treatment for mRCC and multiple targeted therapies, such as tyrosine kinase inhibitors (TKI), mammalian target of rapamycin pathway inhibitors, and vascular endothelial growth factor (VEGF) monoclonal antibody, have been approved as first-line systemic treatments for mRCC [4–6]. More recently, immune-checkpoint inhibitors (ICI) have been provided as a further therapeutic option.

The selection of an appropriate first-line treatment is absolutely crucial, especially because data from the targeted therapy era suggest that only 50% of patients receive second-line treatment, and that only 20% receive third-line treatment [7]. Beyond targeted therapies, various ICI have been tested as novel first-line treatments for mRCC. In recent clinical trials, ICI-based combination therapies including nivolumab plus ipilimumab, atezolizumab plus bevacizumab, pembrolizumab plus axitinib, and avelumab plus axitinib exhibited significant benefits in terms of overall survival (OS) and/or progression-free survival (PFS) benefit for mRCC compared with sunitinib as a standard first-line treatment for mRCC [8–11]. Moreover, updating result of the KEYNOTE-426 trial was recently reported [12]. However, there are scant direct comparative data between these agents to inform optimal treatment decisions and guideline recommendations. Therefore, a systematic review was conducted in all clinical trials assessing first-line systemic therapy of mRCC with sunitinib as the control arm, and network meta-analyses were also conducted to indirectly compare the efficacy and safety of the first-line treatment options.

## Materials and methods

The protocol has been registered in the International Prospective Register of Systematic Reviews database (PROSPERO: CRD42020170483).

### Search strategy

The systematic review and network meta-analysis of phase III randomized controlled trials (RCT) comparing at least two first-line systemic therapies for mRCC (with sunitinib monotherapy as the control arm) were conducted according to the preferred reporting items for systematic reviews and meta-analyses (PRISMA) extension statement for network meta-analysis [13]. A completed PRISMA 2009 checklist was used to describe the methodology of our study (Supplementary Table 1). The PubMed, Web of Science, and Scopus databases were searched to identify reports published until June 2020 in first-line systemic therapy for mRCC. The following keywords were used in our search strategy: (renal cell carcinoma OR renal cell cancer OR kidney carcinoma OR kidney cancer) AND (metastatic OR advanced) AND (Randomized). The primary outcome of interest was OS and PFS, and the secondary outcomes were objective response, and adverse events (AEs). Initial screening was performed independently by two investigators based on the titles and abstracts of the article to identify ineligible reports. Reasons for exclusions were noted. Potentially relevant reports were subjected to a full-text review, and the relevance of the reports was confirmed after the data extraction process. Disagreements were resolved via consensus with a separate committee of investigators.

### Inclusion and exclusion criteria

Studies were included if they investigated metastatic clear-cell RCC patients (Patients) who had undergone systemic therapy as first-line treatment (Intervention) compared with those treated with sunitinib as first-line treatment (Comparison) to assess the differential effects on PFS, OS, objective response, and AEs (Outcome) in phase III randomized studies only. We excluded observational studies, reviews, letters, editorials, replies from authors, case reports, and articles not published in English. In cases of multiple publications on the same cohort, either the higher quality or the most recent publication was selected. References of all papers included were scanned for additional studies of interest. As the focus of this study was the first-line efficacy of these agents in patients who had no history of systemic therapy, studies involving patients who had a history of systemic therapy or studies in which this subset could not be excluded from the overall cohort were excluded from this analysis. As TKIs are widely accepted as the standard of care, studies were excluded if they included interferon or placebo as the control arms. Studies were included only if they involved patients who received sunitinib 50 mg as the control arm.

### Data extraction

Two investigators independently extracted the following information from the included articles: first author’s name, publication year, period of patient recruitment, number of patients, treatment dosage, age, sex, study design, risk group, component of RCC, oncologic outcomes, and AE outcomes. Subsequently, the hazard ratios (HR) and 95% confidence intervals (CI) associated with PFS and OS, objective response rate, and AE rate were retrieved. All HRs were derived from Cox models. All discrepancies regarding data extraction were resolved by consensus with the committee of investigators.

### Risk of bias assessment

The “risk-of-bias” (RoB) evaluation of each study was assessed according to The Cochrane Collaboration's tool for assessing risk of bias [14]. This tool assesses selection bias (random sequence generation and allocation concealment), performance bias, detection bias, attrition bias, reporting bias, and other sources of bias (Supplementary Fig 1). The RoB of each study was assessed independently by two authors. Disagreements were resolved by consultation with the coauthors.

### Statistical analyses

PFS was defined as the time from randomization to the first radiographic progression or death due to any cause. Objective response was defined as the proportion of enrolled and randomly assigned patients who achieved the best response of complete response (CR) or partial response based on investigator assessment. For each outcome, we conducted network meta-analysis using random and fixed-effect models with a Bayesian approach for the direct and indirect treatment comparisons with sunitinib as the common comparator arm [15, 16]. In the assessment for PFS and OS, contrast-based analyses were applied with estimated differences in the log HR and the standard error calculated from the published HR and CI [17]. The relative treatment effects were presented as HR and 95% credible interval (CrI) [15]. With regard to PFS and OS, subgroup analyses were conducted among: intermediate/poor-risk disease and favorable-risk disease defined according to the Memorial Sloan Kettering Cancer Center or International mRCC Database Consortium risk categorization [18, 19]. For the assessment of the objective response and AEs, arm-based analyses were performed to estimate ORs of the objective response and AEs (and 95% CrI) from the available raw data presented in the selected manuscripts [15]. We also estimated the relative ranking of the different treatments for each outcome using the *P* score, which can be considered a frequentist analog to the surface under the cumulative ranking curves [20, 21]. Network plots were utilized to illustrate the connectivity of the treatment networks in terms of OS, PFS, and AEs. Heterogeneity was assessed using *I*^2^ when more than one trial was available for a given comparison. All statistical analyses were performed using R and Stata/MP 14.2 (Stata Corp., College Station, TX); statistical significance was set at *P* < 0.05.

## Results

### Study selection and characteristics

Our initial search identified 4116 publications, and after the elimination of duplicates, a total of 3667 publications were available. A total of 3611 articles were excluded after screening the titles and abstracts, and a full-text review was performed for 56 articles (Supplementary Figure S1). Based on the selection criteria, we identified 6 articles comprising 5297 patients for the systematic review and network meta-analysis [8–12, 22–24]. Extracted data from the six studies are outlined in Tables 1, S2. All these studies were published between 2013 and 2019 and included 2568 [male: 1912 (74.5%); age range: 61–62 years] patients treated with sunitinib and 2639 [male: 1895 (71.8%); age range: 60–62 years] patients treated with other systemic agents.

**Table 1:** Study demographics

Author	Year	Treatment	Dosage	Control	Phase	N (*T*)	N (*C*)	Age (*T*/*C*) (median)	Sex (*T*/*C*)	Favorable (*T*/*C*)	Intermediate (*T*/*C*)	Poor (*T*/*C*)	Component
Rini	2019	Atezolizumab plus bevacizumab	A 200 mg, B 15 mg/kg	S 50 mg	3	454	461	62/60	318/350	89/90	311/318	54/53	CC or Sa
Rini	2016	IMA901 plus sunitinib	I 4.13 mg, S 50 mg	S 50 mg	3	204	135	61/60	142/88	56/35	145/96	3/4	CC
Motzer	2013	Pazopanib	800 mg	S 50 mg	3	557	553	61/62	398/415	151/152	322/328	110/85	CC
Plimack	2020	Pembrolizumab and axitinib	P 200 mg, Ax 5 mg	S 50 mg	3	432	429	62/61	308/320	138/131	238/246	56/52	CC or Sa
Motzer	2019	Nivolumab plus ipilimumab	N 3 mg/kg, Ip 1 mg/kg	S 50 mg	3	550	546	62/62	413/395	125/124	334/333	91/89	CC or Sa
Motzer	2019	Avelumab plus axitinib	Av 10 mg/kg, Ax 5 mg	S 50 mg	3	442	444	62/61	316/344	96/100	283/293	51/45	CC

*A* Atezolizumab, *Av* avelumab, *Ax* axitinib, *B* bevacizumab, *C* control, *CC* clear cell, *I* IMA901, *Ip* ipilimumab, *N* nivolumab, *P* pembrolizumab, *S* sunitinib, *Sa* sarcomatoid, *T* treatment

### Network meta-analysis

The networks of eligible comparisons were graphically represented in network plots in terms of PFS (Supplementary Fig. 2a), OS (Supplementary Fig. 2b), and high-grade AEs (Supplementary Fig. 2c).

### Progression-free survival

**Fig. 1:**
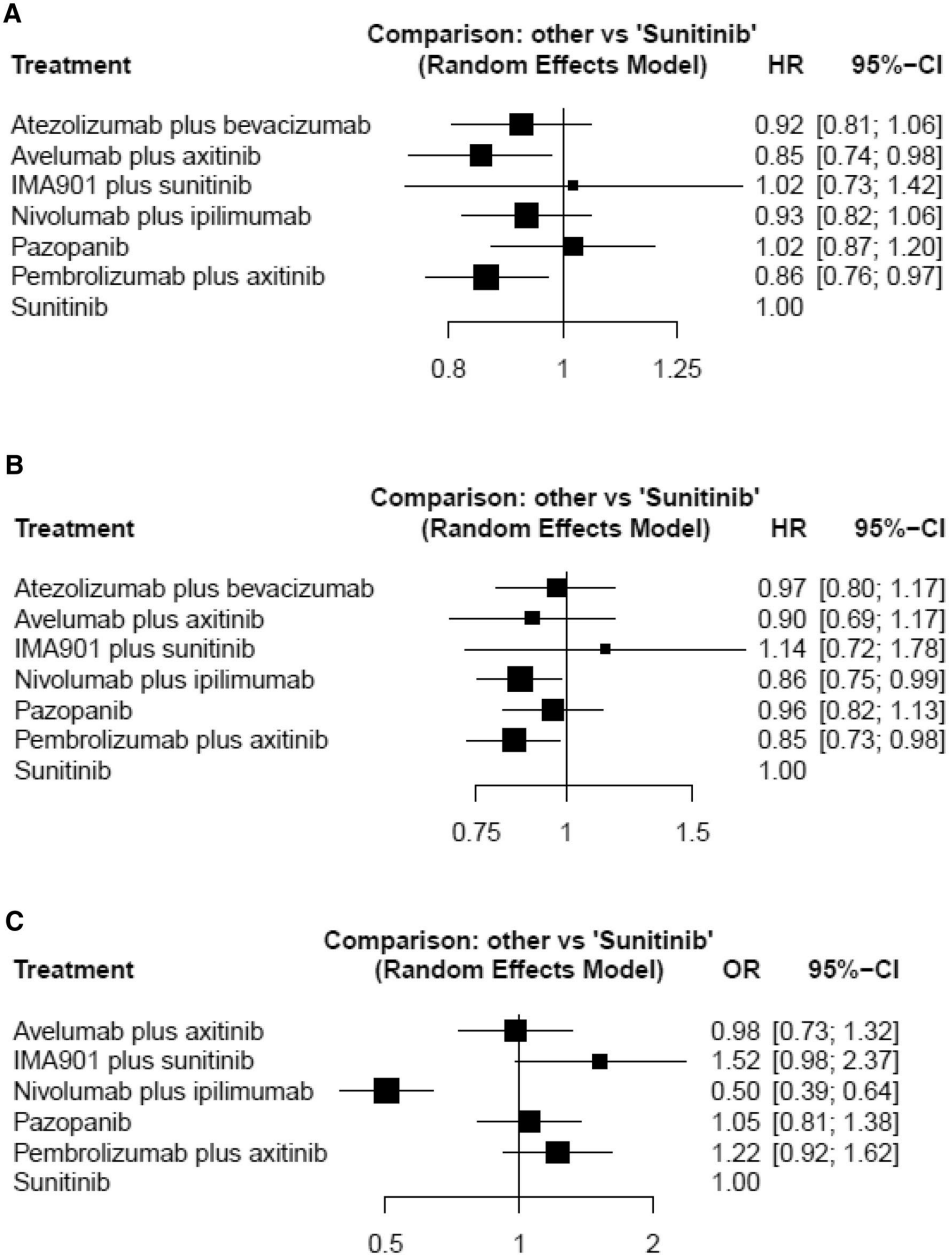
Forest plots showing the association of systemic therapy in metastatic renal cell carcinoma. **a** progression-free survival (PFS), **b** overall survival (OS), **c** adverse event (AE)

A network meta-analysis of seven different agents was conducted for the primary outcome of PFS. Compared with sunitinib, avelumab plus axitinib and pembrolizumab plus axitinib resulted in significantly improved PFS (HR 0.85, 95% CrI 0.74–0.98 and HR 0.86, 95% CrI 0.76–0.97, respectively) (Fig. 1a). Based on analysis of the treatment ranking, avelumab plus axitinib had the highest likelihood of providing the maximal PFS (*P* score: 0.8255) (Table 2). Pembrolizumab plus axitinib was likely to be similarly deemed as the preferred treatment choice (*P* score: 0.8022).

**Table 2:** Analysis of the treatment ranking

Treatment	*P* score (fixed)	*P* score (random)
Progression free survival		
Avelumab plus axitinib	0.8255	0.8255
Pembrolizumab plus axitinib	0.8002	0.8002
Atezolizumab plus bevacizumab	0.5679	0.5679
Nivolumab plus ipilimumab	0.5316	0.5316
IMA901 plus sunitinib	0.3122	0.3122
Sunitinb	0.2377	0.2377
Pazopanib	0.2249	0.2249
Overall survival		
Pembrolizumab plus axitinib	0.8052	0.8052
Nivolumab plus ipilimumab	0.7625	0.7625
Avelumab plus axitinib	0.6141	0.6141
Pazopanib	0.433	0.433
Atezolizumab plus bevacizumab	0.4094	0.4094
Sunitinb	0.2721	0.2721
IMA901 plus sunitinib	0.2038	0.2038
Adverse events		
Nivolumab plus ipilimumab	0.9999	0.9999
Sunitinb	0.5985	0.5985
Avelumab plus axitinib	0.5944	0.5944
Pazopanib	0.4817	0.4817
Pembrolizumab plus axitinib	0.2517	0.2517
IMA901 plus sunitinib	0.0737	0.0737

### Overall survival

A network meta-analysis of seven different agents was conducted for the primary outcome of OS. Compared with sunitinib, nivolumab plus ipilimumab, and pembrolizumab plus axitinib resulted in significantly improved OS (HR 0.86, 95% CrI 0.75–0.99 and HR 0.85, 95% CrI 0.73–0.98, respectively) (Fig. 1b). Based on analysis of the treatment ranking, pembrolizumab plus axitinib had the highest likelihood of providing the maximal OS (*P* score: 0.8052) (Table 2). Nivolumab plus ipilimumab was likely to be similarly deemed as the preferred treatment choice (*P* score: 0.7625).

### Adverse events

Rates of grade 3 ≧ AEs were examined as a measure of toxicity of treatment. A network meta-analysis of six different agents was conducted for the outcome of serious AEs. Compared with sunitinib, nivolumab plus ipilimumab (OR 0.50, 95% CrI 0.39–0.64) was associated with a significantly lower likelihood of toxicity (Fig. 1c). Based on analysis of the treatment ranking, it was highly likely that nivolumab plus ipilimumab had the lowest rate of serious AEs (*P* score: 0.9999) (Table 2).

### Objective response

**Fig. 2:**
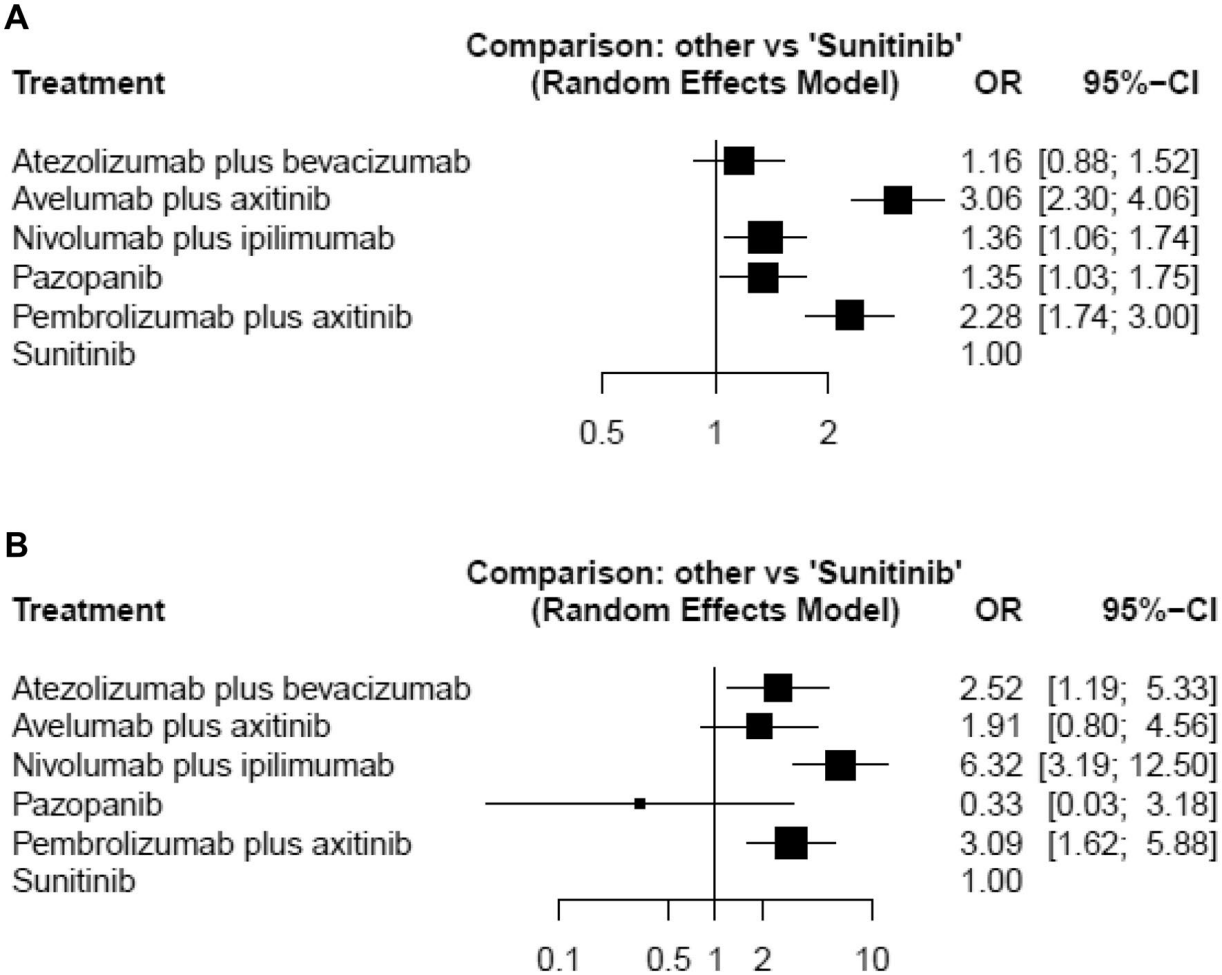
Forest plots showing the association of systemic therapy in metastatic renal cell carcinoma. **a** Objective response, **b** complete response

A network meta-analysis of six agents was performed for the outcome of objective response rates. Compared with sunitinib, avelumab plus axitinib and nivolumab plus ipilimumab and pazopanib, and pembrolizumab plus axitinib resulted in significantly higher objective response rates (Fig. 2a). Based on analysis of the treatment ranking, it was highly likely that avelumab plus axitinib had the highest objective response rate (*P* score: 0.9855), closely followed by pembrolizumab plus axitinib (*P* score: 0.8132) (Supplementary Table 2).

### Complete response

A network meta-analysis of six agents was conducted for the outcome of CR rates. Compared with sunitinib, atezolizumab plus bevacizumab and nivolumab plus ipilimumab, and pembrolizumab plus axitinib resulted in significantly higher CR rates (Fig. 2b). Based on analysis of the treatment ranking, it was highly likely that Nivolumab plus ipilimub had the highest CR rate (*P* score: 0.9742), followed by pembrolizumab plus axitinib (*P* score: 0.6998) (Supplementary Table 2).

### Intermediate/poor-risk subgroup

Based on analysis of the treatment ranking, in patients with intermediate/poor-risk mRCC, pembrolizumab plus axitinib had the highest likelihood of providing the maximal OS (*P* score: 0.8220), closely followed by nivolumab plus ipilimumab (*P* score: 0.7677). Based on Bayesian analysis and analysis of the treatment ranking, avelumab plus axitinib had the highest likelihood of providing the maximal PFS (*P* score: 0.7582), closely followed by pembrolizumab plus axitinib (*P* score: 0.7293) (Supplementary Fig. 3 and Supplementary Table 3).

### Favorable-risk subgroup

Based on analysis of the treatment ranking, in patients with favorable-risk mRCC, IMA901 plus sunitinib had the highest likelihood of providing the maximal OS (*P* score: 0.6136). Based on Bayesian analysis and analysis of the treatment ranking, avelumab plus axitinib had the highest likelihood of providing the maximal PFS (*P* score: 0.8480) (Supplementary Fig. 3 and Supplementary Table 3).

## Discussion

A systematic review was conducted on systemic therapy agents that have been evaluated in RCTs for patients with mRCC in comparison with sunitinib in the first-line setting; we also performed a network meta-analysis and indirectly compared clinically relevant first-line treatment options. This approach generated several important findings. First, pembrolizumab plus axitinib was probably the best treatment option with regard to survival, and statistically more effective than most available treatments. Second, avelumab plus axitinib and nivolumab plus ipilimumab were probably the second best options with regard to survival. Third, nivolumab plus ipilimumab was the best tolerated of all the agents evaluated. The ICI-based combination treatments (nivolumab plus ipilimumab, pembrolizumab plus axitinib, and avelumab plus axitinib) were associated with fewer or similar high-grade AEs than sunitinib.

These developments are of particular interest, as previous network meta-analyses did not include recently reported data and/or analyzed heterogeneous populations [25, 26]. Therefore, we focused only on phase-3 studies which included sunitinib as the control arm and included recently published data, such as updated results from the KEYNOTE-426 trial [12]. As a result, this design may be more reasonable compared with an already published paper by Hahn et al. [25]. In addition, this network meta-analysis included AE outcomes. This is of greater relevance to clinical practice than the recent study by Monteiro et al. [26]. On these points, current paper may more readily facilitate individualized treatment selection.

In this meta-analysis, pembrolizumab plus axitinib, a combination of an anti-programmed death 1 (PD-1) monoclonal antibody and VEGF receptor (VEGFR) TKI, appeared to be the best therapeutic option based on its benefit for OS and PFS. Blockade of immune checkpoints, such as cytotoxic T-lymphocyte-associated antigen 4 (CTLA-4) and PD-1, that are negative regulators which inhibit T-cell proliferation and activity, could result in tumor eradication through reactivation and enhancement of the internal T-cell response [27]; moreover, VEGF inhibition has been shown to suppress angiogenesis as well as increase the recruitment and tumor infiltration of T cells [28, 29]. Indeed, it has been shown in mouse models that simultaneous inhibition of the VEGF and PD-1 pathways not only reduced tumor neovascularization and upregulation of pro-inflammatory cytokines but also inhibited tumor growth [30, 31]. Thus, the finding that the combination of ICI and VEGF axis inhibitors could play a key role in the treatment of mRCC led to a large number of clinical trials to test such combinations. Our pooled analysis of the effects of these combinations revealed that pembrolizumab plus axitinib represented the best treatment with regard to OS and PFS, despite major concerns over toxicity from previous studies combining PD-1 inhibitors nivolumab and pembrolizumab with sunitinib or pazopanib [32, 33]. However, in this analysis, pembrolizumab plus axitinib resulted in similar high-grade AEs to those with sunitinib; this may be because axitinib is a highly selective inhibitor of VEGFR, while sunitinib and pazopanib have a broader range of targets [34].

The use of nivolumab in combination with ipilimumab, a dual checkpoint inhibitor with selective affinity for immune cells that express PD-1 and CTLA-4 molecules, produced impressive results in mRCC patients [8, 22]. While nivolumab plus ipilimumab may not be as effective as pembrolizumab plus axitinib according to our network meta-analysis, this combination was superior to sunitinib, both with regard to PFS/OS and its safety profile, as it was associated with fewer serious AEs. Thus, nivolumab plus ipilimumab appears to provide the most favorable-risk/benefit treatment.

Despite the comprehensive nature of the systematic review undertaken, there are some limitations that need to be considered. First, although indirect treatment comparison analyses have been used and validated for comparing outcomes from RCTs, this approach falls short of a head-to-head treatment comparison. Thus, direct well-designed comparative trials are required to validate the findings of this study. Second, this network meta-analysis was based on the reporting quality of the trials we reviewed and may have been affected by several types of bias, thus limiting the validity of the overall findings. Third, the patient characteristics may have differed significantly between the studies, limiting the comparability of the trials evaluated. Moreover, the OS benefits of the treatments were not evaluated in some of the trials that assessed PFS as the primary endpoint, which did not allow the comprehensive evaluations of OS benefits of all existing treatments. While not being an individual patient data meta-analysis, this study demonstrated no major differences in the patient characteristics, suggesting that some confounding factors, such as prognostic risk categories and PD-L1 status, may have influenced the systemic treatment benefit; this was not ascertainable at the individual patient level. Moreover, because of the limitations of published data, performing a meta-analysis of adjusted effect estimates proved to be impossible. Fourth, the doses and methods of administration of each systemic treatment in the studies included may differ from those in real-world clinical practice, and therefore, the efficacy and toxicity may have differed according to the dose and method of administration. Most notably, while individualized dose adjustment of sunitinib has been shown to improve its efficacy and tolerability, the studies included in this analysis employed the standard dose regimen only [35, 36]. Fifth, some of the treatments evaluated here are least likely to reach the clinic and/or remain of only relative interest (e.g., sunitinib + IMA901). Finally, differences in subsequent therapies received across the treatment arms in the trials evaluated may have possibly influenced the OS results. In addition, the OS data from some trials were immature; thus, the study outcomes could change in their final analysis. Nevertheless, the current network meta-analysis suggests that pembrolizumab plus axitinib appears to be the best option in the first-line setting. This could help improve clinical decision making, while the present meta-analysis is not intended to replace the need for head-to-head clinical trials of contemporary first-line therapies.

## Conclusion

In this systematic review and network meta-analysis of first-line systemic therapies for patients with mRCC, based on an indirect comparison of data from phase-3 clinical trials, pembrolizumab plus axitinib was identified as having a high likelihood of providing the maximum PFS and OS benefits. In addition, nivolumab plus ipilimumab appeared to have the most favorable tradeoff between efficacy and tolerability. These findings may provide guidance to patients and clinicians for treatment decisions, when considered with other aspects that drive personalized medicine strategies for mRCC.

### Electronic supplementary material

Below is the link to the electronic supplementary material.

Supplementary file1 (PDF 308 kb)

Supplementary file2 (PDF 41 kb)

Supplementary file3 (PDF 93 kb)

Supplementary file4 (PDF 65 kb)

Supplementary file5 (PDF 199 kb)

Supplementary file6 (PDF 37 kb)

Supplementary file7 (PDF 59 kb)

Supplementary file8 (DOCX 14 kb)
