# Supplementary figures and images for: Systemic therapy for metastatic renal cell carcinoma in the first-line setting: a systematic review and network meta-analysis
Source: Cancer Immunol Immunother. 2020 Aug 5;70(2):265–73. doi: 10.1007/s00262-020-02684-8 (PMC7889529; doi:10.1007/s00262-020-02684-8)

Figure 1

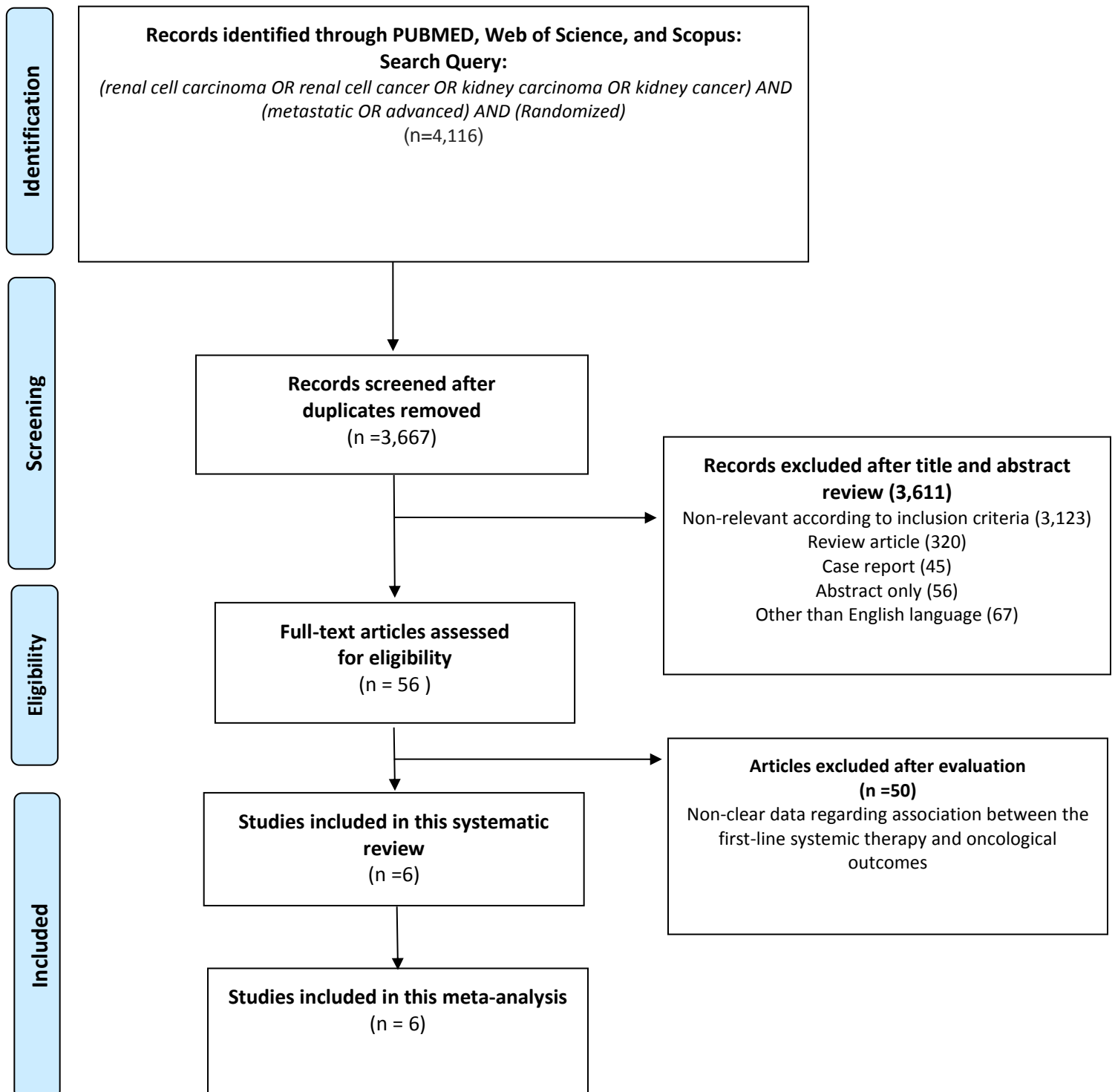

Supplement: Supplementary file 1 — Supplementary file1 (PDF 308 kb) [file 262_2020_2684_MOESM1_ESM.pdf]
