# Supplementary material for: Systemic therapy for metastatic renal cell carcinoma in the first-line setting: a systematic review and network meta-analysis
Source: Cancer Immunol Immunother. 2020 Aug 5;70(2):265–73. doi: 10.1007/s00262-020-02684-8 (PMC7889529; doi:10.1007/s00262-020-02684-8)
Supplement: Supplementary file 2 — Supplementary file2 (PDF 41 kb) [file 262_2020_2684_MOESM2_ESM.pdf]

Supplementary Figure 1

Risk of bias summary of the included studies for network meta-analysis

(A) Random sequence generation (selection bias); (B) allocation concealment (selection bias); (C) blinding of participants and personal (performance bias); (D) blinding of outcome assessment (detection bias); (E) incomplete outcome data (attrition bias); (F) selective reporting (reporting bias); (G) other bias. Green circles represent a low risk of bias and confounding, red circles represent a high risk of bias and confounding, and yellow circles represent an unclear risk of bias and confounding.

Author, year	*A*	*B*	*C*	*D*	*E*	*F*	*G*
Rini 2019	+	−	−	−	+	+	+
Plimack 2020	+	?	−	+	+	+	+
Rini 2016	+	−	−	−	+	+	+
Motzer 2013	+	?	−	−	+	+	+
Motzer 2019 (CheckMate214)	+	?	−	−	+	+	+
Motzer 2019(JAVELIN Renal 101)	+	?	−	+	+	+	+
