# Supplementary material for: Systemic therapy for metastatic renal cell carcinoma in the first-line setting: a systematic review and network meta-analysis
Source: Cancer Immunol Immunother. 2020 Aug 5;70(2):265–73. doi: 10.1007/s00262-020-02684-8 (PMC7889529; doi:10.1007/s00262-020-02684-8)

Supplementary Figure 2

Network plots showing the association of systemic therapy in metastatic renal cell carcinoma

A. Progression free survival

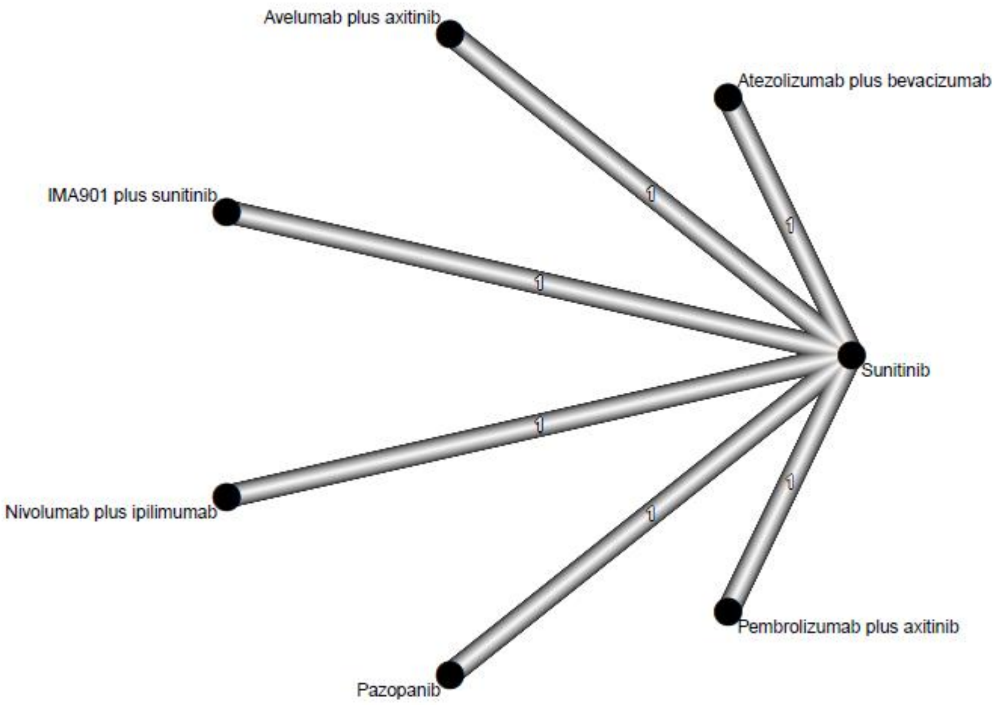

B. Overall survival

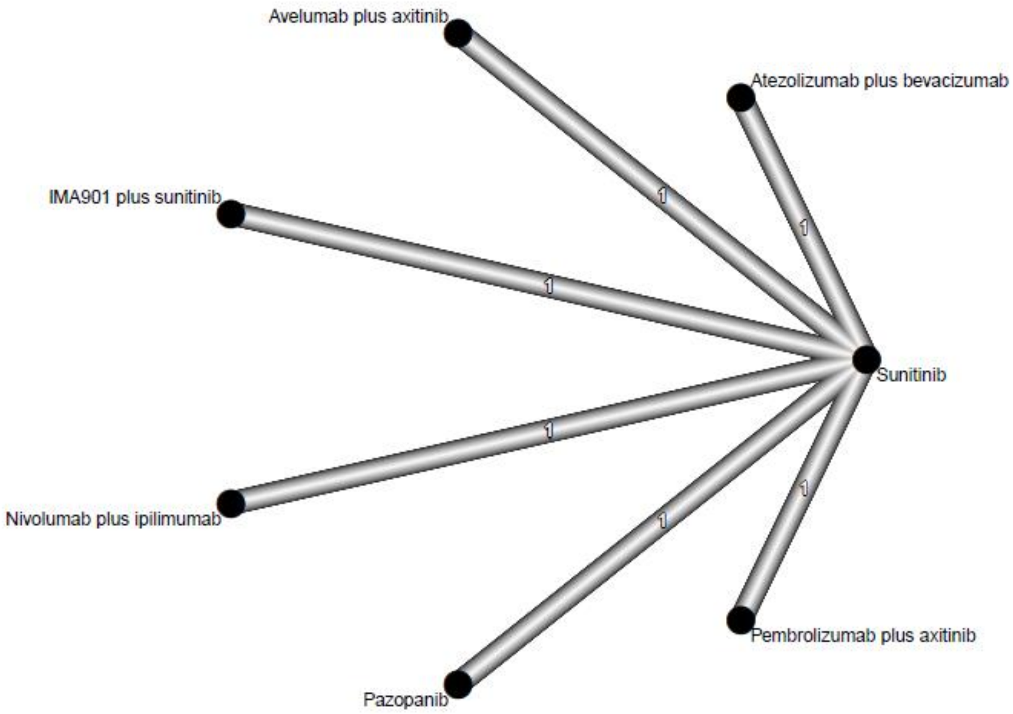

C. Adverse event

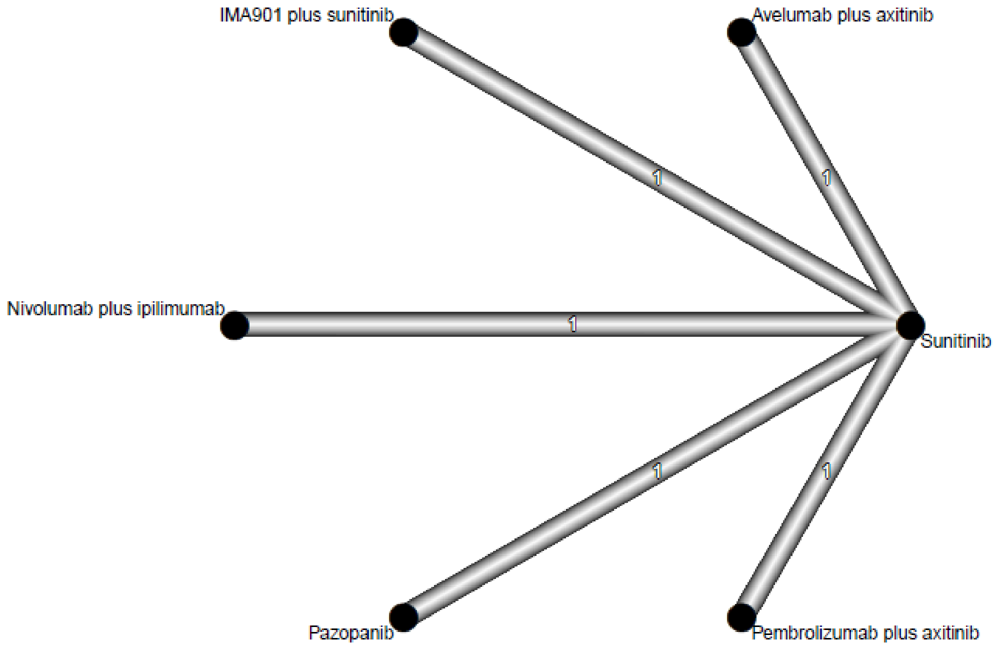

Supplement: Supplementary file 3 — Supplementary file3 (PDF 93 kb) [file 262_2020_2684_MOESM3_ESM.pdf]
