# Supplementary material for: Systemic therapy for metastatic renal cell carcinoma in the first-line setting: a systematic review and network meta-analysis
Source: Cancer Immunol Immunother. 2020 Aug 5;70(2):265–73. doi: 10.1007/s00262-020-02684-8 (PMC7889529; doi:10.1007/s00262-020-02684-8)

Supplementary Figure 3

Forest plots

A. Overall survival (Intermediate/poor-risk)

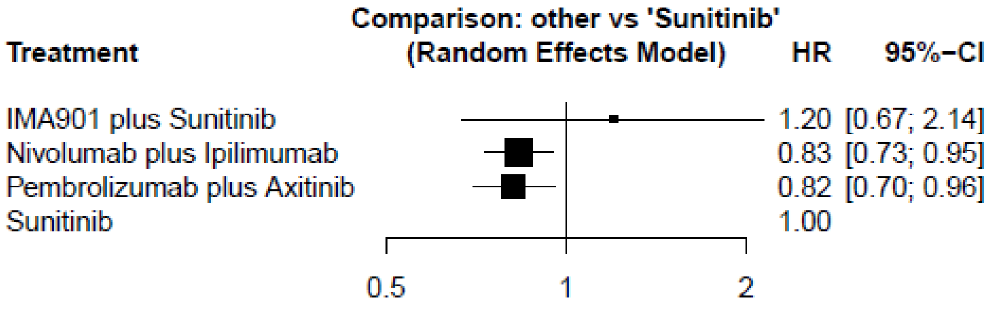

B. Progression free survival (Intermediate/poor-risk)

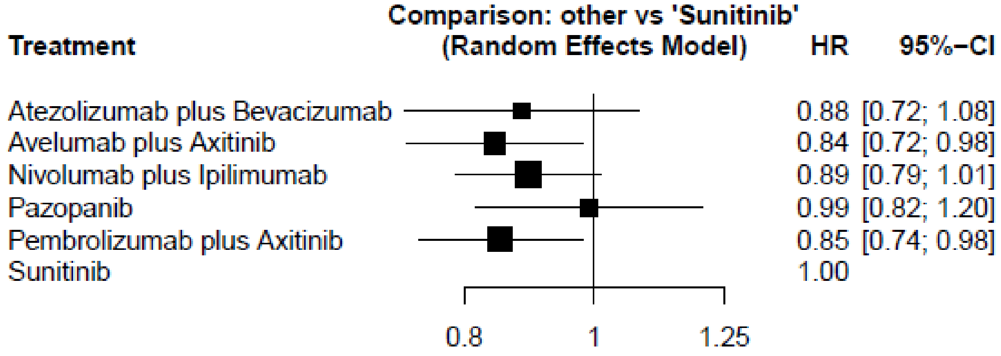

C. Overall survival (favorable-risk)

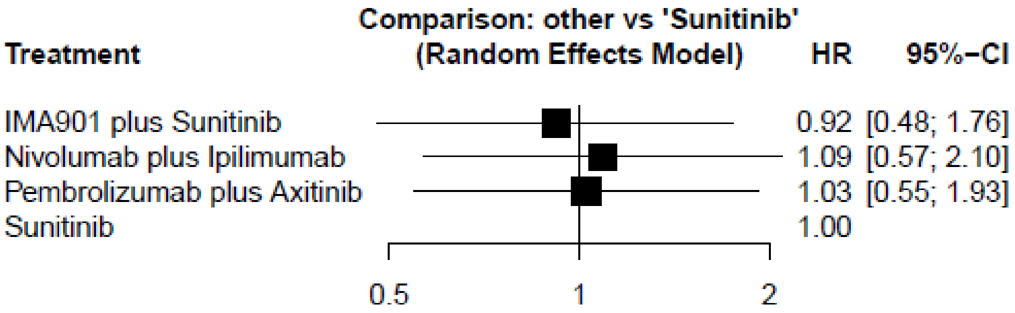

D. Progression free survival (favorable-risk)

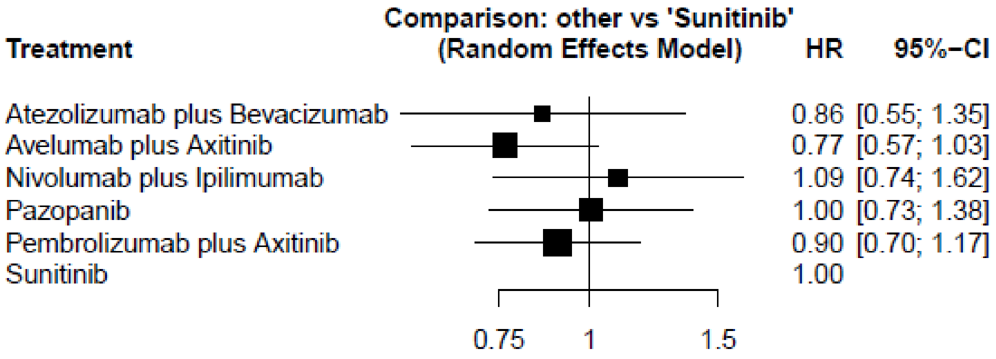

Supplement: Supplementary file 4 — Supplementary file4 (PDF 65 kb) [file 262_2020_2684_MOESM4_ESM.pdf]
