# Supplementary material for: Systemic therapy for metastatic renal cell carcinoma in the first-line setting: a systematic review and network meta-analysis
Source: Cancer Immunol Immunother. 2020 Aug 5;70(2):265–73. doi: 10.1007/s00262-020-02684-8 (PMC7889529; doi:10.1007/s00262-020-02684-8)
Supplement: Supplementary file 6 — Supplementary file6 (PDF 37 kb) [file 262_2020_2684_MOESM6_ESM.pdf]

Supplementary Table 2 Analysis of the treatment ranking

Objective response		
Treatment	P score (fixed)	P score (random)
Avelumab plus axitinib	0.9855	0.9855
Pembrolizumab plus axitinib	0.8132	0.8132
Nivolumab plus ipilimumab	0.4645	0.4645
Pazopanib	0.4486	0.4486
Atezolizumab plus bevacizumab	0.2556	0.2556
Sunitinb	0.0326	0.0326
Complete response		
Treatment	P score (fixed)	P score (random)
Nivolumab plus ipilimumab	0.9742	0.9742
Pembrolizumab plus axitinib	0.6998	0.6998
Atezolizumab plus bevacizumab	0.601	0.601
Avelumab plus axitinib	0.4763	0.4763
Sunitinb	0.1822	0.1822
Pazopanib	0.0665	0.0665
