# Supplementary material for: Systemic therapy for metastatic renal cell carcinoma in the first-line setting: a systematic review and network meta-analysis
Source: Cancer Immunol Immunother. 2020 Aug 5;70(2):265–73. doi: 10.1007/s00262-020-02684-8 (PMC7889529; doi:10.1007/s00262-020-02684-8)
Supplement: Supplementary file 7 — Supplementary file7 (PDF 59 kb) [file 262_2020_2684_MOESM7_ESM.pdf]

Supplementary Table 3 Analysis of the treatment ranking

Intermediate / poor risk					
Progression free survival			Overall survival		
Treatment	P score (fixed)	P score (random)	Treatment	P score (fixed)	P score (random)
Avelumab plus axitinib	0.7582	0.8317	Pembrolizumab plus axitinib	0.822	0.822
Pembrolizumab plus axitinib	0.7293	0.6402	Nivolumab plus ipilimumab	0.7677	0.7677
Atezolizumab plus bevacizumab	0.5911	0.567	Sunitinb	0.2465	0.2465
Nivolumab plus ipilimumab	0.5657	0.4106	IMA901 plus sunitinib	0.1638	0.1638
Pazopanib	0.2274	0.3953			
Sunitinb	0.1282	0.3572			
Favorable risk					
Progression free survival			Overall survival		
Treatment	P score (fixed)	P score (random)	Treatment	P score (fixed)	P score (random)
Avelumab plus axitinib	0.848	0.848	IMA901 plus sunitinib	0.6136	0.6136
Atezolizumab plus bevacizumab	0.6265	0.6265	Sunitinb	0.5103	0.5103
Pembrolizumab plus axitinib	0.5792	0.5792	Pembrolizumab plus axitinib	0.4752	0.4752
Pazopanib	0.3656	0.3656	Nivolumab plus ipilimumab	0.4009	0.4009
Sunitinb	0.3396	0.3396			
Nivolumab plus ipilimumab	0.2411	0.2411			
