# Supplementary material for: Systemic therapy for metastatic renal cell carcinoma in the first-line setting: a systematic review and network meta-analysis
Source: Cancer Immunol Immunother. 2020 Aug 5;70(2):265–73. doi: 10.1007/s00262-020-02684-8 (PMC7889529; doi:10.1007/s00262-020-02684-8)
Supplement: Supplementary file 8 — Supplementary file8 (DOCX 14 kb) [file 262_2020_2684_MOESM8_ESM.docx]

Table 2 Study outcomes

| Author | Year | Treatment | OS median (T/C) | PFS median (T/C) | CR (T) | CR (C) | OR (T) | OR (C) | AE G3≧ (T) | AE G3≧ (C) |
| --- | --- | --- | --- | --- | --- | --- | --- | --- | --- | --- |
| Rini | 2019 | Atezolizumab plus bevacizumab | 33.6/34.9 | 11.2/8.4 | 24/454 (5.3%) | 10/461 (2.2%) | 166/454 (36.6%) | 153/461 (33.2%) | NR | NR |
| Rini | 2016 | IMA901 plus sunitinib | 33.17/NRE | 15.22/15.12 | NR | NR | NR | NR | 116/202 (57.4%) | 62/132 (47.0%) |
| Motzer | 2013 | Pazopanib | 28.4/29.3 | 8.4/9.5 | 1/557 (0.2%) | 3/553 (0.5%) | 171/557 (30.7%) | 137/553 (24.8%) | 412/554 (74.4%) | 402/548 (73.4%) |
| Plimack | 2020 | Pembrolizumab and axitinib | NRE/35.7 | 15.4/11.1 | 38/432 (8.8%) | 13/429 (3.0%) | 260/432 (60.2%) | 171/429 (39.8%) | 287/429 (66.9%) | 265/429 (62.4%) |
| Motzer | 2019 | Nivolumab plus ipilimumab | NRE/37.9 | 9.7/9.7 | 58/550 (10.5%) | 10/546 (1.8%) | 227/550 (41.3%) | 186/546 (34.1%) | 250/547 (45.7%) | 335/535 (62.6%) |
| Motzer | 2019 | Avelumab plus axitinib | NRE/NRE | 13.8/8.4 | 15/442 (3.4%) | 8/444 (1.8%) | 227/442 (51.4%) | 114/444 (25.7%) | 309/434 (71.2%) | 314/439 (71.5%) |
| Abbreviation: AE (adverse event), C (control), CR (complete response), OR (objective response), OS (overall survival), PFS (progression free survival), NR (not reported), NRE (not reached), T(treatment) | | | | | | | | | | |
